# Public Contribution in Qualitative Research With Parents of Children Treated for Cancer: Description of and Reflections on a Collaborative Data Analysis Approach

**DOI:** 10.1111/hex.70586

**Published:** 2026-02-01

**Authors:** Ella Thiblin, Christina Reuther, Mattias Bergqvist, Tho Huynh, Johan Lundgren, Sandra Rösler, Joanne Woodford, Louise von Essen

**Affiliations:** ^1^ Department of Women's and Children's Health CIRCLE—Complex Intervention Research in Health and Care, Uppsala University Uppsala Sweden; ^2^ ENGAGE Study Public Contribution Group Uppsala Sweden; ^3^ Department of Health, Medicine and Caring Sciences, Division of Nursing Sciences and Reproductive Health Norrköping Linköping University Linköping Sweden

**Keywords:** childhood cancer, parents, public contribution in research, qualitative analysis

## Abstract

**Background:**

Collaborative data analysis (CDA) in qualitative research is an approach for working with public contributors as co‐researchers in analysing data. Different approaches have been outlined in a Best Practice Framework for CDA: (1) consultation; (2) development; (3) application; and (4) development and application. Four characteristics of successful CDA are also presented; that the CDA process is: (1) co‐produced; (2) realistic within available time and resources; (3) manageable for public contributors; and (4) effective in handling group dynamics and expectations. We adopted a development and application approach to CDA to analyse data from a qualitative study embedded within the single‐arm feasibility trial ENGAGE, exploring the acceptability and feasibility of a cognitive behavioural therapy intervention (EJDeR) for parents of children treated for cancer, and study procedures.

**Objectives:**

Overall aim: to describe and reflect on the CDA approach used. Specific objectives: to (1) describe the CDA approach used; (2) reflect on the CDA approach used and map reflections onto characteristics of successful CDA; (3) summarise the potential impact of the CDA approach used on findings; and (4) report the costs of the public contribution activities.

**Methods:**

Three public contributors collaborated with three research team members to analyse 36 qualitative interviews using the Framework Method. Public contributors received training and supervision, completed all analysis steps, independently developed coding frameworks, applied them to the data and interpreted findings. Together with research team members, they took part in a workshop to reflect on the CDA approach used, with reflections mapped onto four characteristics of successful CDA.

**Findings:**

In the reflection workshop, the CDA approach was described as including the characteristics of successful CDA, for example, that public contributors handled demands of the process well, and that relationships and power dynamics were well managed. Potential impact on findings included public contributors bringing in nuances to the analysis overlooked by research team members, for example, their analysis highlighted that participants in ENGAGE experienced difficulties differentiating between EJDeR and ENGAGE study procedures. Public contributors and research team members categorised data in slightly different ways. The total cost for public contribution activities was ≈21,963 EUR.

**Conclusion:**

The CDA approach used extends the Best Practice Framework for CDA by providing an example of CDA adopting a development and application approach. Our description of the CDA approach used may inform other researchers interested in CDA, and contributes to ongoing conversations about embedding ethical and equitable public contribution throughout the research lifecycle.

**Patient or Public Contribution:**

Public contributors and research team members contributed to the reflections described and writing, editing, and reviewing the manuscript.

## 1 Background

Public contribution in research refers to the involvement of members of the public, including patients or caregivers, as active research partners throughout the research lifecycle [1]. With this approach, research is conducted in collaboration *with* patients and/or members of the public, rather than *about* or *for* them [2]. Public contribution is suggested to enhance accountability, relevance and transparency of research by ensuring the perspectives of those most affected by the outcomes are integrated throughout the research process [3, 4]. This may result in less research waste, for example, by identifying relevant research questions [5], and improving recruitment [6] and retention [7, 8] into clinical research. The value of public contribution is increasingly recognised across academic disciplines, particularly in health research, where it is promoted in research guidance such as the UK Medical Research Council's (MRC) framework for developing and evaluating complex interventions [9]. Furthermore, public contribution is emphasised by major research funders such as the National Institute for Health and Care Research (NIHR) in the United Kingdom, the Research Council of Norway and the Swedish Research Council for Health, Work and Welfare [10].

Public contribution can be integrated at any stage of the research lifecycle, from identifying research questions and designing studies, to analysing data and disseminating findings [11]. However, public contribution is more commonly embedded in early stages of the research lifecycle [12, 13, 14, 15]. For example, collaborative priority‐setting exercises, such as those facilitated by the James Lind Alliance's Priority Setting Partnerships, are established methods for identifying research questions that reflect real‐world concerns [16]. Public contribution in later stages of the research lifecycle, including data collection and data analysis, remains less consistent and may be more challenging to implement [12, 13, 15]. Nevertheless, interest is growing, both within the Nordic countries and internationally, in engaging public contributors in all stages of the research lifecycle [15]. When applied to qualitative analysis, public contribution has been shown to enhance the credibility, depth and real‐world relevance of findings [13, 15, 17]. However, descriptions of public contribution in qualitative research are often lacking [18], and costs for public contribution are rarely reported.

### 1.1 Public Contribution Framework

Several frameworks have been proposed to guide good practice in public contribution [11]. One such framework is the Best Practice Framework for Collaborative Data Analysis (CDA) in qualitative mental health research (hereafter referred to as *The Best Practice Framework for CDA*), suggested by Jennings et al. [13] (see Table 1). The framework authors conducted a critical literature review of papers on CDA in qualitative health research, identifying approaches and characteristics associated with effective CDA. Findings were synthesised and developed into a CDA methodology in collaboration with public contributors. Thereafter, the CDA methodology was piloted and refined within the context of a qualitative mental health study and presented as the proposed Best Practice Framework for CDA. Four different approaches to CDA (consultation, development, application and development and application), and four characteristics of successful CDA (presented in Table 1) were identified in the review and incorporated into the framework. The final framework was structured in three phases: (1) preparation; (2) CDA: co‐production of a coding framework; and (3) application: Integration, refinement, publication and reflective learning. Action points outlined in the phases of the presented framework focused mainly on consultation and development approaches to CDA. The framework authors suggested a need for future research to extend the framework to the development and application approach. Although originally developed for mental health research, the framework has also informed CDA in other areas such as cultural food security [19] and care in care homes [20].

**Table 1:** Approaches to and characteristics of successful collaborative data analysis in accordance with the best practice framework for collaborative data analysis (CDA).

Approaches to collaborative data analysis (CDA)	
Approach	Description
Consultation	Researchers conduct the analysis and subsequently present the results to public contributors who provide feedback.
Development	Public contributors are involved early in the analysis process and help develop codes, themes, and/or frameworks.
Application	Researchers develop codes, themes, and/or frameworks that are subsequently applied to the data by public contributors.
Development and application	Public contributors receive extensive training in data analysis and are involved in all data analysis steps.

Characteristics of successful CDA	
Characteristic	Description
The process is co‐produced	Public contributors are supported in making interpretations based on the data, while their unique perspectives are considered. Different opinions are valued. When there is disagreement, a synthesis of perspectives is aimed for.
The process is realistic within available time and resources	Sufficient time and financing are allocated for organising and facilitating the CDA process. The number of public contributors and researchers is relatively small.
The demands of the process are manageable for public contributors	Public contributors receive sufficient training, ideally including practical and visual aids to support their learning. Necessary materials are readily accessible, and the CDA process is tailored to the skills and needs of public contributors, enabling them to succeed and supporting their ability to work with the task.
Group expectations and dynamics are effectively managed	The responsibilities of public contributors and the expected time commitment are clearly communicated. Roles and task responsibilities are well defined, with awareness of potential power imbalances. All involved are also recognised as having multiple identities that extend beyond the labels of ‘public contributor’ and ‘researcher.’

### 1.2 Context: The ENGAGE Feasibility Trial

In this article, we describe and reflect on the CDA approach used to analyse data from a qualitative study [21] embedded within the single‐arm feasibility trial ENGAGE [22]. Following the feasibility phase of the MRC framework for developing and evaluating complex interventions [9], ENGAGE aimed to examine the acceptability and feasibility of an internet‐administered low‐intensity cognitive behavioural therapy (CBT) intervention (EJDeR) for parents of children treated for cancer, and the ENGAGE study procedures. We conducted a qualitative interview study embedded in ENGAGE to explore the acceptability and feasibility of the intervention and study procedures from the perspective of participating parents, to inform refinements and modifications to the intervention and study procedures [21]. We collaborated with public contributors, adopting a development and application approach to CDA [13], to analyse data from the qualitative interview study. We did not prospectively follow the Best Practice Framework for CDA [13] in planning the CDA approach used. Instead, we used the framework retrospectively to structure our reflections on the CDA approach used, with particular attention to the four characteristics of successful CDA. Findings from the qualitative study are reported elsewhere [21].

### 1.3 Aims and Objectives

The overall aim was to describe and reflect on the CDA approach used when analysing interviews exploring the acceptability and feasibility of EJDeR and ENGAGE study procedures. Objectives were to (1) describe the CDA approach used; (2) reflect on the CDA approach used and map reflections onto characteristics of successful CDA as outlined in the Best Practice Framework for CDA; (3) summarise the potential impact of the CDA approach used on findings from the qualitative study, as perceived by public contributors and research team members; and (4) report the costs of the public contribution activities.

## 2 Methods

Reporting adheres to the Guidance for Reporting Involvement of Patients and the Public (GRIPP 2) checklist [23], see File S1.

### 2.1 ENGAGE Public Contribution Group

At the end of ENGAGE, participating parents were asked via e‐mail to express interest in collaborating with the research team. Those who had expressed interest in collaboration were e‐mailed about the opportunity to contribute to our planned CDA in the qualitative study and informed about the approximate timeline and reimbursement details. Four public contributors were recruited. Three contributed to all steps of the CDA approach, and one withdrew after Workshop 3 (see Table 2 for a description of all data analysis steps). The three public contributors who completed all steps of the CDA approach were two fathers (M.B. and T.H.) and one mother (S.R.) of children previously treated for cancer who resided in different areas of Sweden. No specific inclusion criteria for public contributors, apart from having participated in ENGAGE, were applied, and they had no prior experience in qualitative research.

### 2.2 ENGAGE Research Team Members

Research team members who conducted the qualitative analysis included two female PhD students (E.T., MSc Psychology and licensed psychologist; and C.R., MSc Public Health), and one male researcher (J.L., PhD Nursing Sciences, Associate Professor in Nursing Sciences). All had prior experience in qualitative data analysis. Additional research team members included one female principal investigator (L.v.E., Professor in Healthcare Sciences and licensed psychologist) and one female researcher (J.W., PhD in Psychology, Associate Professor in Caring Sciences), both experienced in qualitative methods. L.v.E. and J.W. provided peer examination of findings and supervised E.T. and C.R.

### 2.3 The CDA Approach Used

Public contributors worked as co‐researchers in all data analysis steps, and the CDA approach used was in accordance with a development and application approach, as outlined in the Best Practice Framework for CDA [13].

The description of the CDA approach we used is informed by a data analysis plan developed by E.T., C.R., L.v.E., and J.W., and a data analysis log kept by E.T. We conducted an inductive content analysis using the Framework Method [24], with coding at the semantic level, that is, focusing on the explicit content of interviews rather than interpreting latent meanings or assumptions. Analysis followed a structured five‐step procedure [24], conducted separately by public contributors and research team members to minimise the risk of public contributors' voices being lost, a common issue when researcher and public contributor analyses are merged [25]. The choice of the data analysis method (i.e., the Framework Method) was guided by its appropriateness for both experienced and inexperienced researchers [24] to minimise imbalances in experience and knowledge between public contributors and research team members. The Framework Method and CDA approach used are presented in Table 2.

**Table 2:** The framework method and the collaborative data analysis approach used.

Analysis step	Description	Activities conducted by public contributors and research team members
I. Familiarisation – Reading, re‐reading, and understanding the data.	Transcripts read multiple times to gain in‐depth familiarity with the data.	Public contributors divided 36 transcripts between them. Research team members divided 52 transcripts between them^a^.
II. Development of Initial Coding Framework – Coding and developing a working analytical framework^b^.	Transcripts independently and inductively coded in two coding cycles: (1) initial coding and (2) refinement, consolidation, and reduction of codes. Codes collaboratively reviewed, merged, renamed, and grouped into broader categories during coding and categorisation workshops to develop a working analytical framework.	Public contributors coded transcripts by hand. Research team members coded transcripts using NVivo (version 1.7.1). Research team members facilitated public contributors’ coding workshops. Public contributors were not present at research team members’ workshops.
III. Indexing – Systematically applying codes and categories to the data.	Transcripts individually indexed (i.e., working analytical framework applied). Iterative revisions to the analytical framework made and salient quotations identified during indexing workshops.	Public contributors indexed transcripts by hand. Research team members indexed transcripts using NVivo. Research team members facilitated public contributors’ indexing workshops. Public contributors were not present at research team members’ workshops.
IV. Charting – Summarising data into matrices.	Indexed data charted into matrices, with one matrix developed per category, using Microsoft Excel. Each matrix included columns representing codes and rows summarising data for each transcript, including illustrative quotations.	Research team members charted the public contributors’ indexed data, given that public contributors were not using NVivo. Research team members charted indexed data using NVivo.
V. Mapping and Interpretation – Analysing similarities and differences within and across cases, drawing conclusions from data.	Data mapped and interpreted by (1) describing content within each category; (2) identifying patterns within and across cases; (3) refining and synthesising interpretations; and (4) summarising key findings in written memos during mapping and interpretation workshops.	Research team members facilitated public contributors’ mapping and interpretation workshops. Public contributors were not present at research team members’ workshops.

^a^ Public contributors did not analyse their own interviews or those of other public contributors, or those assigned to the withdrawing public contributor.

^b^ This step is presented as two separate steps (‘coding’ and ‘developing a working analytical framework’) in the original Gale et al. [24] paper.

Upon completing the data analysis steps outlined in Table 2, written memos developed in analysis step V (Mapping and Interpretation) were used to inform manuscript writing for the qualitative study [21], including synthesising categories into overarching key areas, led by research team members (E.T. and J.W.). The manuscript was reviewed and edited by public contributors and research team members.

### 2.4 Public Contribution Activities

To facilitate the CDA, we conducted 11 workshops with public contributors between August 2022 and April 2023, see Table 3. Between workshops, public contributors engaged in individual work (i.e., reading, coding and indexing transcripts).

**Table 3:** Overview of public contribution activities.

Activity	Type of activity	Description	Location	Length
Workshop 1^a^ August 2022	Introduction and training	Research team members introduced public contribution in research, qualitative research, and the planned CDA approach.	Online via Zoom	3 h
Individual work	Reading transcripts (Step I. Familiarisation)	Public contributors read transcripts multiple times to gain in‐depth familiarity with the data.	N/A	Unknown^b^
Workshop 2 October 2022	Coding: Training and application (Step II. Development of Initial Coding Framework)	Research team members introduced the qualitative analysis method used (Framework Method [24]. Public contributors made an initial coding of transcripts (coding cycle: initial coding).	Stockholm, Sweden	6 h
Individual work	Coding (Step II. Development of Initial Coding Framework)	Public contributors continued coding transcripts (coding cycle: initial coding).	N/A	Unknown^b^
Workshop 3 October 2022	Coding: Continued application (Step II. Development of Initial Coding Framework)	Research team members provided training on common challenges in coding and ways of overcoming them. Research team members and public contributors discussed coding challenges experienced by public contributors. Public contributors refined, consolidated, and reduced codes.	Stockholm, Sweden	6 h
Individual work	Coding (Step II. Development of Initial Coding Framework)	Public contributors continued coding transcripts (coding cycle: refinement, consolidation, and reduction of codes).	N/A	Unknown^b^
Workshop 4 January 2023	Categorisation: cInitial category development (Step II. Development of Initial Coding Framework)	Public contributors presented individual sets of refined, consolidated, and reduced codes and collaboratively merged these into one set of codes. Public contributors collaboratively grouped codes into initial categories.	Stockholm, Sweden	6 h
Workshop 5 January 2023	Categorisation: dContinued development (Step II. Development of Initial Coding Framework)	Public contributors collaboratively grouped codes into categories.	Online via Zoom	1.5 h
Workshop 6 February 2023	Categorisation: Finalising (Step II. Development of Initial Coding Framework)	Public contributors collaboratively grouped codes into categories.	Online via Zoom	1.5 h
Individual work	Indexing (Step III. Indexing)	Public contributors indexed transcripts.	N/A	Unknown^b^
Workshop 7 February 2023	Checking and refining framework (Step III. Indexing)	Public contributors discussed and refined the framework.	Online via Zoom	0.5 h
Individual work	Indexing (Step. III. Indexing)	Public contributors continued indexing transcripts.	N/A	Unknown^b^
Workshop 8 March 2023	Checking and refining framework (Step III. Indexing)	Public contributors discussed and refined the framework.	Online via Zoom	0.5 h
Individual work	Indexing (Step III. Indexing)	Public contributors continued indexing transcripts.	N/A	Unknown^b^
Workshop 9 March 2023	Checking and refining framework (Step III. Indexing)	Public contributors discussed and refined the framework.	Online via Zoom	0.5 h
Individual work	Indexing (Step III. Indexing)	Public contributors continued indexing transcripts.	N/A	Unknown^b^
Workshop 10 March 2023	Data interpretation (Step V. Mapping and Interpretation)	Public contributors mapped and interpreted data.	Stockholm, Sweden	6 h
Workshop 11 April 2023	Data interpretation (Step V. Mapping and Interpretation)	Public contributors mapped and interpreted data.	Online via Zoom	0.5 h

^a^ Both public contributors and research team members were present at all workshops.

^b^ Unknown, as not all public contributors reported all their working hours.

^c^ In face‐to‐face workshops, categorisation was aided by printed codes and Post‐it notes.

^d^ In Zoom workshops, categorisation was aided by the digital design tool Canva.

Public contributors were trained in qualitative data analysis by E.T., C.R., and J.L. Training consisted of workshops (face‐to‐face and via Zoom) and optional individual supervision via Zoom. Workshop 1 included an introduction to public contribution in research, basic principles of qualitative data analysis with examples, and coding exercises. Coding exercises consisted of example interview excerpts unrelated to the study topic (written by E.T.) that public contributors first coded individually. Subsequently, coding was discussed, and research team members shared potential codes for the excerpts. Practical information was provided regarding the organisation of public contribution work, including expectations regarding workload, an approximate timeline and reimbursement. Subsequent training was provided through face‐to‐face workshops, addressing challenges encountered with the data analysis procedure, for example, via coding and categorisation exercises. Coding exercises were structured similarly to those in the first training workshop, with public contributors individually coding interview excerpts, followed by discussions and examples from research team members. After the coding exercise, public contributors practised categorising their codes together by grouping them under categories written on post‐it notes. Although the training workshops were not based on a formal or pre‐existing training framework, they were guided by a practice‐based, ‘learning by doing’ approach. Rather than providing extensive theoretical background in qualitative methodology, training focused on worked examples, hands‐on coding exercises and group discussions. A variety of formats were used to support learning, including PowerPoint presentations, group discussions and practical work exercises (e.g., generating categories from Post‐it notes with individual codes).

Initially, it was planned that four public contributors would analyse 48 of 52 transcripts (to maintain impartiality, public contributors did not analyse their own interviews or those of other public contributors). However, one public contributor withdrew early in the CDA process, and their assigned transcripts were removed to avoid overburdening the remaining public contributors. As a result, the three public contributors analysed 36 transcripts in total (a mean of 12 transcripts, with a range of 11–13). All transcripts allocated to public contributors were pseudonymised, with potentially identifying information (e.g., city names, hospitals) redacted. Research team members analysed all 52 transcripts in pseudonymised form (a mean of 17.3 transcripts, with a range of 17–18). The approximate timeline communicated to public contributors during recruitment and training was extended during the analysis to allow sufficient time for all analysis steps, after discussion and approval from all public contributors.

### 2.5 Reflections on the CDA Approach We Used

After all analysis steps were conducted, and findings were reported, all public contributors (M.B., T.H., and S.R.) and two research team members (E.T. and C.R.) held a 2‐h reflection workshop to reflect on the CDA approach used. The workshop was held online via Zoom, and discussions were structured around the topics: (1) information and initial phase of the CDA; (2) supervision and support; (3) group dynamics, roles and communication; and (4) potential impact of CDA on findings from the qualitative study. Public contributors and research team members also had the opportunity to bring up any reflections not covered by these topics. C.R. took detailed notes of workshop discussions and sent these notes to all attendees via e‐mail for comments and amendments. All workshop attendees approved the notes without amendments. Reflections were subsequently mapped onto the Best Practice Framework for collaborative CDA [13] by E.T. and sent to all public contributors and C.R. via e‐mail for review. Comments were subsequently incorporated into the manuscript by ET and circulated via e‐mail again to all workshop attendees and other co‐authors for final approval.

### 2.6 Reimbursement for Public Contribution Activities

Public contributors were paid an hourly rate for public contribution activities, and their travel expenses were covered. Hotel accommodation was provided to facilitate attendance at face‐to‐face workshops. Not all public contributors claimed reimbursement for all their working hours.

### 2.7 Ethical Considerations and Risk Management

The CDA approach used was approved by the Swedish Ethical Review Board (see ethical approval statement).

Public contributors were informed that they could withdraw from the work at any time without having to state a reason, and they were introduced to the project principal investigator (L.v.E.) as a point of contact for any questions or concerns. Although public contributors were not considered study participants and no formal risk protocol was established specifically for the CDA process, support mechanisms were in place to ensure the well‐being of public contributors. The principal investigator (L.v.E.) and one member of the research team (E.T.) are licensed psychologists with experience in identifying and managing distress and unanticipated risks to individuals' well‐being in research contexts, including within ENGAGE. These arrangements ensured that appropriate support and follow‐up could be provided if distress arose, even though no such situations occurred.

## 3 Findings

### 3.1 Reflections on the CDA Approach We Used

Reflections were made on the CDA approach used, areas in which the approach could be improved, and the potential impact of the approach on findings from the qualitative study [21]. All reflections presented were discussed during the reflection workshop and are considered inputs from both public contributors and research team members, unless explicitly stated otherwise (i.e., ‘we’ refers to both public contributors and research team members). Reflections are presented mapped onto the four characteristics of successful CDA in the Best Practice Framework for CDA. The potential impact of public contribution on research findings is summarised, and costs for public contribution activities are reported.

#### 3.1.1 The CDA Process Is Co‐Produced

Reflections were made on whether the CDA approach we used facilitated authentic and genuine collaboration. Public contributors and research team members perceived their respective analyses as equal and that all contributions were respected and valued. Although it was recognised that the public contribution group was relatively homogeneous in terms of education level and age, their diverse personalities and life experiences were described as adding depth and nuance to discussions, and these differences were appreciated.

#### 3.1.2 The CDA Process Is Realistic Within Available Time and Resources

It was discussed that the CDA method was time‐consuming, but it was described as worthwhile for all involved, as motivation was high, and we could see results from everybody's effort. Flexibility in scheduling workshops, as well as the use of both face‐to‐face and online workshops, was considered to facilitate efficient time use. Extending the timeline for public contribution activities was perceived as positive, as public contributors wanted to be involved in all steps. However, public contributors highlighted the importance of presenting such requests (e.g., to extend the timeline) as open questions and not pressure public contributors to take on extra work. Budgeting for face‐to‐face workshops was considered important, as it facilitated learning among public contributors and fostered trust and a working relationship among all involved. The number of public contributors was perceived as appropriate for fostering rich discussions while allowing everyone to contribute actively. However, including one or two additional public contributors was discussed as potentially beneficial for further broadening perspectives, without creating a group that was too large.

Research team members described being mindful that they analysed data as part of their employment, while public contributors worked on the project in addition to their everyday employment. To avoid burdening public contributors with technical demands (e.g., learning to use qualitative data analysis software such as NVivo) and to ensure their contribution was realistic, they were asked to complete coding and indexing by hand. Furthermore, research team members were responsible for transferring handwritten data to NVivo and charting the data, a division of work that was described as functioning well. The location for face‐to‐face workshops was chosen for being easy to travel to, and research team members booked travel and accommodation for public contributors, which was described as facilitating the public contributors' work.

#### 3.1.3 The Demands of the CDA Process Are Manageable for Public Contributors

Public contributors found training and supervision helpful, particularly in the early stages when coding felt unfamiliar and challenging. However, research team members avoiding positioning themselves as ‘experts’ occasionally led to uncertainty among public contributors about whether they were ‘doing it right’. The training procedure, with a ‘learning by doing’ approach, was appreciated by public contributors, but also contributed to their initial uncertainty about their coding and categorisation. Over time, public contributors described their confidence growing, primarily through group discussions where different interpretations were welcomed and clarified collaboratively.

Public contributors noted they had all completed university‐level education and were accustomed to collaborative work. Both public contributors and research team members reflected that high demands were placed on public contributors regarding language and reading skills, and abstract reasoning, which was discussed as possibly not being suitable for all potential public contributors. We reflected on alternative, potentially more inclusive approaches. For example, working with public contributors using different CDA approaches (e.g., consultation or development), or in different phases of the analysis, for example, some public contributors join at the indexing step, or provide feedback on codes and/or categories, depending on their preference.

#### 3.1.4 Group Expectations and Dynamics Are Effectively Managed

Public contributors understood what was expected of them, both in their overall role and in specific tasks. However, we reflected that roles evolved over time, as the CDA procedure was new for both public contributors and research team members. A particular challenge, described by research team members, was navigating dual roles, for example, analysing the data and providing training and supervision to public contributors. Efforts by research team members to avoid influencing public contributors' interpretations during workshops were perceived as sometimes resulting in public contributors feeling unsure about the quality of their analysis. Despite this, it is possible that research team members may have unintentionally steered public contributors' analysis. We discussed that one potential way to overcome this challenge could be to involve external researchers in the CDA approach, thereby reducing potential bias. However, this would come at the cost of losing contextual knowledge, and it was discussed as being beneficial that research team members could answer questions about EJDeR and ENGAGE to help public contributors understand interview content. Public contributors also held dual roles in this project, serving as both public contributors and former ENGAGE participants. Public contributors' situated knowledge (i.e., of the study context and personal experience) was perceived as beneficial for understanding the interview content. However, we reflected that there is a risk that public contributors' own knowledge may influence their analysis and interpretation when they are also study participants. In our CDA approach, public contributors found group discussions and collective reflections helpful strategies for balancing their experiential perspectives with a more distanced, outsider lens.

Group dynamics were described as respectful and supportive, with no single individual dominating the discussions. It was acknowledged that research team members and public contributors held multiple identities besides ‘researcher’ and ‘parent of a child treated for cancer’, respectively, and individual personalities and skills were appreciated.

We found it important to acknowledge that research team members had access to information about the public contributors, as public contributors were former ENGAGE participants and had participated in the interviews themselves. Research team members made efforts to be transparent about how information was handled (e.g., that they read transcripts of the public contributors' interviews, but in pseudonymised form). The relationship between public contributors and research team members was described as professional, and spending time with one another was considered a way of building trust. Public contributors reflected that their previous professional experience of working on complex group activities may have contributed to group dynamics, for example, being able to navigate group discussions and disagreements effectively.

### 3.2 The Potential Impact of the CDA Approach We Used on Research Findings

Similarities and differences in the analyses by public contributors and research team members are published [21]. In summary, the analyses were similar overall, which strengthened confidence in the findings among both public contributors and research team members. However, we reflected that similarities in analysis also led to overlap. Public contributors mentioned that a synthesis of findings after conducting separate analyses could have been interesting to present. Although there were similarities, public contributors added nuances to the findings that the research team had overlooked. We reflected this may be due to differences in perspectives and experiences between public contributors and research team members (e.g., being study participants vs*.* researchers in ENGAGE). An example of a difference in nuance is that public contributors identified an important consideration regarding participants' understanding of study information presented in ENGAGE, namely that intervention research is not well known among the public, and being invited to participate in a ‘study’ may be interpreted as answering questions, for example, in a survey, rather than working with an intervention. Furthermore, public contributors analysed data about ENGAGE study participants' experiences of EJDeR (i.e., the intervention) and study procedures (e.g., eligibility interview and data collection) together (e.g., under the category ‘General experiences with participation’). Conversely, research team members analysed data concerning the intervention and study procedures separately (e.g., under categories ‘Appreciated parts of the intervention’ and ‘Acceptability of study procedures’). This indicates ENGAGE participants may have perceived the EJDeR intervention and the ENGAGE procedures as a ‘package’, which has implications for how to formulate questions to explore the acceptability and feasibility of interventions and study procedures. Using prompts to clarify what a question concerns, for example, the intervention or a study procedure, may help in future research. Additionally, research team members tended to use academic terminology when analysing data, for example, by focusing on mechanisms of impact. In addition to its potential impact on research findings, the CDA process used provided both public contributors and research team members with a valuable learning experience that will benefit the planning, execution and evaluation of future public contribution projects.

### 3.3 Costs for Public Contribution Activities

Public contributors were paid an hourly rate of 270 SEK (≈25.48 EUR). The total cost for public contribution activities was ≈21,963 EUR. Public contributors spent ≈585 h in workshops (including travel time) and reported individual work between workshops, with a total cost of ≈14,903 EUR. Other costs included travel expenses (≈1282 EUR), hotel accommodation (≈1716 EUR), venue costs for face‐to‐face workshops, lunches and refreshments during face‐to‐face workshops (≈ 4062 EUR).

## 4 Discussion

We have described the CDA approach used and mapped reflections on it to the characteristics of successful CDA outlined in the Best Practice Framework for CDA [13]. We have also summarised the potential impact of the CDA approach used on findings, and reported the costs of public contribution activities. The public contribution we adopted was aligned with the development and application approach outlined in the Best Practice Framework for CDA. Reflections suggested the CDA approach used included characteristics of successful CDA, as outlined in the Best Practice Framework for CDA [13]. We reflected that CDA had a potential impact on the findings; for example, we developed a more nuanced understanding of how participants may have interpreted the study information and how the intervention and study procedures are perceived as a ‘package’ rather than as separate processes. The total cost for public contribution activities was ≈21,963 EUR.

The CDA approach used, which included public contributors in all data analysis steps, may have placed greater demands on public contributors in comparison to other CDA approaches in which contributors act as consultants [26] or take part in only some data analysis steps [20]. Specifically, public contribution activities required time commitment as well as reading and abstract reasoning skills. The challenge of balancing meaningful contribution with reasonable demands on public contributors is well‐documented in previous research using CDA [27, 28, 29]. Opinions vary on what constitutes reasonable demands on public contributors; some recommend involving contributors primarily in analytical conversations rather than coding data [29], while others advocate involving contributors in all data analysis steps [30]. One argument for more flexible opportunities for public contribution is that appropriate approaches may differ between individuals, and opportunities should exist for public contributors with different skills, capacities and interests [31]. In the reflection workshop, we discussed that offering flexible roles could potentially enable a wider range of contributors to contribute meaningfully to future research using CDA. Conducting CDA using a development and application approach may lessen the risk of tokenistic public contribution, but may result in the CDA process not being inclusive. Labelling this level of public contribution as the ‘gold standard’, as suggested in the Best Practice Framework for CDA [13], is potentially problematic if it excludes interested individuals. However, CDA using a development and application approach does not necessarily mean public contributors need to analyse the same amount of data as research team members. For example, in the CDA approach we used, we reduced the number of transcripts for the public contributor group due to practical circumstances (i.e., one public contributor withdrawing), and there are other examples in the wider literature of similar adjustments [27, 32]. A related challenge is that authorship criteria for scientific publications require contributors to review and approve the final manuscript [33]. In non‐English‐speaking contexts, such as Sweden, this requirement can be a barrier where English proficiency varies. Future research, including public contributors in writing for publication, could consider offering translation support or bilingual facilitation to ensure contributors are not excluded from authorship opportunities.

Relational aspects and power dynamics, central concepts in the public contribution literature [34, 35, 36], received relatively little attention in our reflections on the CDA approach we used. This is of particular concern given that relational aspects play a crucial role in ensuring the ethical standard of public contribution work [37]. Maintaining ethical standards includes maintaining professional boundaries and avoiding assumptions that public contributors are seeking to build personal relationships [38], while still developing trusting relationships over time, as this is critical in fostering equality between researchers and public contributors [37]. We recognised that it was possible to foster professional, trusting working relationships. We perceived face‐to‐face workshops as facilitating relationship building, as reported in the wider literature [37]. Clear communication at the outset of CDA about the nature and focus of the work public contributors are expected to do can help manage expectations (e.g., by establishing that the focus is on data analysis rather than support or the sharing of personal experiences) and ensure mutual understanding [36]. Explicit agreements on boundaries and roles could further support trust and clarity in future CDA approaches.

The emotional demands put on public contributors are acknowledged in the literature [34, 39], but were not perceived as a challenge in the CDA approach we used. This may be due to the nature of our public contribution activities. For example, emotional exhaustion among public contributors has been found to result from sharing personal experiences [37], which was not part of the CDA approach used. While we recognise researchers should be aware of the potential emotional demands placed on public contributors [35], it should not be assumed that public contributors will react emotionally to a greater extent than research team members. Qualitative research on sensitive topics can evoke distressing emotions in anyone involved [40, 41], and access to appropriate supervision (e.g., including debriefing after emotionally taxing analysis steps [41]) is important for both public contributors and researchers.

### 4.1 Methodological Considerations

There are some considerations regarding the CDA approach we used that should be raised. First, conducting two parallel data analyses allowed public contributors' voices to be perceived as having equal weight with those of research team members, potentially enhancing the credibility of the findings [42]. However, it also led to repetition in the main findings paper [21]. Reporting two separate analyses was challenging when structuring the findings, as the same content was sometimes reported in one category in one analysis and across multiple categories in the other, resulting in content overlap. The final report writing was researcher‐led, primarily for practical reasons (e.g., time and resource constraints), and a more collaborative writing process may have enabled further synthesis of findings. Alternatively, a ‘whole team approach’ (e.g., initial data analysis steps done separately, followed by ‘whole‐team’ meetings to co‐create the analytical framework) could have minimised overlap, albeit potentially at the cost of losing public contributors' voices [43]. Second, costs for the public contribution activities in the CDA approach used are challenging to evaluate, as they are rarely reported and may vary between projects due to contextual factors (e.g., differences in medium wages and travel expenses across countries). However, during the reflection workshop, we discussed that the CDA approach used was resource‐intensive in terms of time, which in turn required significant financial resources. We wanted public contributors to be able to take part regardless of geographical location, and travel and accommodation costs were a substantial part of the budget. We acknowledge that not all projects have the same resources as we did, and projects with more restricted budgets may consider recruiting public contributors living within commuting distance to facilitate face‐to‐face meetings. We conducted public contribution activities over an 8‐month period, which provided public contributors with sufficient time for analysis; however, this may not be feasible in projects with shorter deadlines. Going forward, developing adaptable CDA timelines and budget models could make similar public contribution activities feasible in more resource‐limited projects. Third, public contributors holding a dual role as both study participants and public contributors is debated in the literature [44]. While the well‐established definition of patient and public involvement by NIHR does not include study participants [2], it has been noted that it may be helpful to involve former study participants as public contributors in intervention studies, as they have unique insights into the intervention that is being developed or evaluated [44]. We found that public contributors' experiences of the intervention deepened their understanding of the interviews analysed. The importance of paying attention to how one's own experiences may influence data analysis has been acknowledged in previous research, both in relation to public contributors [32] and academic researchers [32, 45]. We found that continuous reflective discussions helped both public contributors and research team members become aware of their own preconceptions and assumptions. Based on our experience, we would recommend other researchers to consider inviting former study participants to CDA in the context of intervention research, and to plan for ongoing, reflexive discussions around different perspectives and potential influences on interpretation throughout the process.

### 4.2 Ethical Considerations

The ENGAGE was ethically approved, and additional approval for conducting the CDA approach reported in this article was obtained from the Swedish Ethical Review Board. In the Swedish context, as well as internationally [46], it is not always clear which public contribution activities require ethical approval. As public contributors are considered research partners, and not study participants [47], researchers seeking ethical approval may inadvertently introduce power inequalities into public contribution activities [46]. We considered it appropriate to obtain ethical approval for our CDA process, since public contributors were also ENGAGE participants, and moved from the role of study participants to research partners. The reflections workshop was viewed as a collaborative process in which public contributors and researchers reflected together in equal roles and was not considered a research activity involving the collection and analysis of data; that is, reflections came from both public contributors and researchers, and reporting was a joint effort among all involved. While our reflections informed the findings presented here, reflections were not considered research data, and thus were not analysed nor intended to produce transferable findings.

### 4.3 Strengths and Limitations

We mapped our experiences onto the Best Practice Framework for CDA, as outlined by Jennings et al. [13]. However, our reflections are anecdotal, and the mapping was done retrospectively. There is a lack of evaluations of public contribution [48], and future research should aim to evaluate its impact, for example, the impact of working with CDA [49, 50]. Potential evaluation methods include impact logs and/or qualitative interviews [51, 52, 53]. In addition, the use of reflections could have been strengthened by adopting structured reflection models such as Gibbs' cyclical model of reflection [54]. With Gibb's model, structured questions are asked, which could include a description of public contribution, feelings around public contribution, evaluation and analysis of public contribution experiences, conclusions and action plans [54]. Using a structured reflection model could have prompted public contributors and research team members to cover additional topics in the reflection workshop and facilitated a clearer action plan for future public contribution activities. For instance, a targeted discussion about the implications of the dual role of study participant and public contributor on group and power dynamics could have been a valuable contribution to the literature on public contribution. Furthermore, the reflections presented are from research team members and three public contributors who conducted all CDA steps; it is possible that the public contributor who withdrew from the process may have reflected on the approach used differently.

Despite limitations, this article has several strengths. First, public contributors are co‐authors, and findings are informed by reflections from both public contributors and research team members. Second, this article extends the Best Practice Framework to describe CDA using a development and application approach. In our view, although resource‐intensive, this approach enriched the analysis by ensuring that public contributors' perspectives were integrated at every stage. Finally, the CDA approach we used provided a learning experience and may offer guidance for others seeking to use CDA.

## 5 Conclusions

We have described the CDA approach we used to involve public contributors in a qualitative study embedded in ENGAGE and presented our reflections on the CDA approach used, mapped onto characteristics of successful CDA outlined in the Best Practice Framework for CDA [13]. Furthermore, we reflected on the potential impact of CDA on research findings and reported the costs for public contribution activities. We aimed for meaningful and structured public contribution throughout all stages of the analysis. We reflected that using a development and application approach in accordance with the Best Practice Framework for CDA facilitated genuine collaboration and mutual learning between public contributors and research team members.

While the CDA approach we used was resource‐intensive, it proved to be feasible. Importantly, our reflections highlight key tensions in CDA work, particularly the balance between meaningful contribution and inclusivity. CDA using a development and application approach may reduce tokenism and enhance the authenticity of public contribution, but it also places high demands on public contributors that may not be reasonable for all individuals. Future research should continue to further develop CDA approaches tailored to individual backgrounds, interests and preferences. We hope this article provides researchers with guidance on using CDA and contributes meaningfully to ongoing conversations about developing methods to facilitate equitable public contribution throughout the research lifecycle.

## Author Contributions

**Ella Thiblin:** conceptualisation, methodology, investigation, writing – original draft, visualisation, project administration. **Christina Reuther:** investigation, writing – review and editing, project administration. **Mattias Bergqvist:** investigation, writing – review and editing. **Tho Huynh:** investigation, writing – review and editing. **Johan Lundgren:** investigation, writing – review and editing. **Sandra Rösler:** investigation, writing – review and editing. **Joanne Woodford:** methodology, writing – original draft, project administration, supervision. **Louise von Essen:** conceptualisation, methodology, writing – review and editing, resources, project administration, supervision, funding acquisition.

## Ethics Statement

The ENGAGE feasibility trial was approved by the Regional Ethical Review Board in Uppsala, Sweden (Dnr: 2017/527), and conducted in accordance with the Declaration of Helsinki, ensuring the welfare and rights of all participants, as well as Good Clinical Practice (GCP) guidelines. An ethical amendment was obtained to involve parents (public contributors) in data analysis from the Swedish Ethical Review Authority on 13‐06‐2022, ref: 2022–02779‐02.

## Conflicts of Interest

The authors declare no conflicts of interest.

## Supporting information

Supporting file 1_GRIPP 2 Checklist.

## Data Availability

Data sharing is not applicable to this article as no datasets were generated or analysed during the current study.
